# The Dual Role of the Pervasive “Fattish” Tissue Remodeling With Age

**DOI:** 10.3389/fendo.2019.00114

**Published:** 2019-02-26

**Authors:** Maria Conte, Morena Martucci, Marco Sandri, Claudio Franceschi, Stefano Salvioli

**Affiliations:** ^1^Department of Experimental, Diagnostic and Specialty Medicine (DIMES), University of Bologna, Bologna, Italy; ^2^Interdepartmental Centre “L. Galvani” (CIG), University of Bologna, Bologna, Italy; ^3^Venetian Institute of Molecular Medicine, Padova, Italy; ^4^Department of Biomedical Sciences, University of Padova, Padova, Italy; ^5^IRCCS Istituto delle Scienze Neurologiche di Bologna, Bologna, Italy; ^6^Lobachevsky State University of Nizhny Novgorod, Nizhny Novgorod, Russia

**Keywords:** adipose tissue, aging, lipid deposition, organ involution, inflammaging

## Abstract

Human aging is characterized by dramatic changes in body mass composition that include a general increase of the total fat mass. Within the fat mass, a change in the proportions of adipose tissues also occurs with aging, affecting body metabolism, and playing a central role in many chronic diseases, including insulin resistance, obesity, cardiovascular diseases, and type II diabetes. In mammals, fat accumulates as white (WAT) and brown (BAT) adipose tissue, which differ both in morphology and function. While WAT is involved in lipid storage and immuno-endocrine responses, BAT is aimed at generating heat. With advancing age BAT declines, while WAT increases reaching the maximum peak by early old age and changes its distribution toward a higher proportion of visceral WAT. However, lipids tend to accumulate also within lipid droplets (LDs) in non-adipose tissues, including muscle, liver, and heart. The excess of such ectopic lipid deposition and the alteration of LD homeostasis contribute to the pathogenesis of the above-mentioned age-related diseases. It is not clear why age-associated tissue remodeling seems to lean toward lipid deposition as a “default program.” However, it can be noted that such remodeling is not inevitably detrimental. In fact, such a programmed redistribution of fat throughout life could be considered physiological and even protective, in particular at extreme old age. In this regard, it has to be considered that an excessive decrease of subcutaneous peripheral fat is associated with a pro-inflammatory status, and a decrease of LD is associated with lipotoxicity leading to an increased risk of insulin resistance, type II diabetes and cardiovascular diseases. At variance, a balanced rate of fat content and distribution has beneficial effects for health and metabolic homeostasis, positively affecting longevity. In this review, we will summarize the present knowledge on the mechanisms of the age-related changes in lipid distribution and we will discuss how fat mass negatively or positively impacts on human health and longevity.

## Introduction

Aging is a complex process characterized by progressive changes in body mass composition that lead to a functional decline at cellular and organ levels over time. With advancing age, lean mass and bone mineral density decrease, while total fat mass increases and changes its distribution, particularly in the abdominal region, often without concomitant changes in body mass index (BMI) (1). In mammals, fat mass accumulates as adipose tissue or ectopic lipid deposition. Adipose tissue is a dynamic organ involved in the regulation of energy homeostasis, mainly divided in three types, brown (BAT), white (WAT), and BEIGE which differ in embryogenesis, anatomy, and function (2–4). While BAT possesses high levels of mitochondria and is specialized in fat burning to generate heat, WAT is characterized by a low density of mitochondria and it is generally involved in lipid storage in two biological distinct compartments: subcutaneous (SAT) and visceral (VAT) adipose tissue. WAT is not only involved in the storage of lipids, but also plays an important role as immuno-endocrine organ (5). With advancing age, BAT mass declines, while WAT increases reaching the maximum peak by early old age and changing its distribution toward a higher proportion of VAT (2). WAT redistribution is also accompanied by an accumulation of fat mass in non-adipose tissues and organs, such as muscle, liver, heart, pancreas and others, that normally contain only small amounts of fat, stored within lipid droplets (LDs) (6). Adipose tissue shows also an extraordinary plasticity (7), in fact it can differentiate into another type of adipose tissue, such as BEIGE (8, 9) or replace the parenchyma of organs that undergo involution with age, such as the thymus.

It is well-described that increased proportions of fat mass affect body metabolism and play a central role in many chronic diseases, including insulin resistance, obesity, cardiovascular diseases, type II diabetes, and sarcopenia (1, 10, 11).

In this review, we will summarize the changes that occur in lipid distribution with increasing age, and we will propose that: (i) the generalized increased amount of fat (in form of adipose tissue or intracellular lipid droplets) should be interpreted as an adaptive response to environmental conditions and, as such, is not *per se* a detrimental phenomenon; (ii) it can be a case of antagonistic pleiotropy, *i.e*., while it has detrimental effects at old age, it can turn to be protective in extreme longevity.

## The Role of Fat Mass in the Evolution and During AGING

Body fat storage has a long evolutionary history and represents a fundamental strategy to store energy fuel that is crucial for survival in conditions where food is not continuously available (12). All living organisms, from prokaryotes to mammals, have the ability to store energy that can be mobilized in response to a need, such as growth, metabolism, and reproduction (13). While simple organisms obtain and use energy only in response to an immediate need, more complex organisms have developed a mechanism to store energy in form of adipose tissue or ectopic lipid deposition (5). In insects, fat bodies represent not only lipid deposits but also organs able to perform complex endocrine and exocrine functions similar to those of the liver (14). Fat bodies play their biosynthetic and metabolic activities by the production of circulating proteins, acting as hormones, necessary in several physiological conditions, such as morphogenesis, egg maturation, and lipid and carbohydrate metabolism (15).

In mammals, fat mass distribution has reached a quite high degree of complexity. In these organisms, fat mass is widely distributed in the whole body and it is involved in many physiological processes, *i.e.*, energy supply during periods of starvation or undernutrition, regulation of metabolic homeostasis, reproduction, thermoregulation, immune response with production of cytokines and chemokines (13, 16). However, fat mass does not remain constant during lifespan, but changes in content and distribution from birth to extreme old age (17). These changes regard both adipose tissue and intracellular lipid stores (LDs) in non-adipose sites, and some of them, as in the case of thymic involution occurring at puberty, have to be considered as physiologically programmed. Much less is known about the fat mass changes occurring at advanced age. The “thrifty genotype theory” (18) explains the accumulation of adipose tissue as a strategy of metabolic adaptation, shaped by natural selection, to survive conditions of food scarcity. However, the existence of a specific genetic program leading to lipid deposition during aging is unlikely, considering that, according to the more advanced theories, aging in mammals is neither programmed nor selected by evolution. Thus, it seems more plausible that the age-associated lipid deposition is rather a phenomenon of adaptive remodeling in response to environmental conditions. Like many other adaptive phenomena it is conceivable that it may have both beneficial and detrimental effects. A growing body of evidence demonstrates that high levels of fat mass are associated with the development of several metabolic diseases (10), for this reason very often the fat deposition is considered *tout court* purely detrimental for the organism, and every age-associated weight gain bad for health. However, several lines of evidence demonstrate that also the deficiency of adipose tissue, as observed in transgenic mice or during lipodystrophy, results in the development of metabolic dysfunctions (19–21). For example, human lipodystrophies are characterized by genetic defects in lipid storage with total or partial loss of fat mass and related metabolic abnormalities such as insulin resistance and hypertension (22). Moreover, transgenic mice expressing dominant-negative protein A-ZIP/F (19) are characterized by the absence of WAT, reduced amount of BAT, elevated serum glucose, insulin, free fatty acids (FFA), triglycerides and type II diabetes (19). Consistently, adipose tissue results to have beneficial protective effects toward metabolic syndromes (19, 23–25). We argue that also during aging fat deposition can have beneficial effects, in particular at very old age, when it can be an important reserve of strategic energy crucial for resilience and recovery from stress and, eventually, for survival.

## Fat Mass Distribution in Human Aging

In normal aging total fat mass increases over the adult lifespan with a peak at about 65–70 years, while in extreme old age fat mass decreases (26). In the following paragraphs, we will describe the different types of body fat mass (adipose tissue and ectopic lipid depots) and the re-distribution of such fat mass during aging.

### White Adipose Tissue (WAT)

WAT is a complex tissue composed by unilocular adipocytes, other cellular types, such as immune and stem cells, and connective tissue (4). The main role of WAT is the storage of energy and, as such, it actively controls the energy metabolism of all organs and tissues. In fact WAT secretes cytokines and proteins that communicate with other organs, such as brain, liver, muscle, or pancreas (27). As mentioned in the introduction, the two major WAT depots are SAT and VAT. In human body, SAT is present in the hypodermis of abdominal, gluteal, and femoral districts, while the counterpart VAT resides within abdominal cavity (omental, mesenteric, retroperitoneal, gonadal fat) and mediastinum. Moreover, VAT is also present around specific organs such as heart, stomach and blood vessels. With aging WAT increases and re-distributes, in particular a relative decline of SAT in the abdomen and limb region (thigh, calve), and a concomitant increase of VAT can be appreciated (4, 28). While SAT is considered protective, being the main source of adiponectin (29), VAT is considered detrimental as it produces pro-inflammatory mediators such as leptin, that boost the status of chronic, subclinical inflammation typically found in the elderly and indicated as inflammaging (30). Aging also entails a dysregulation in WAT and a consequent excess of circulating FFA. Higher levels of circulating FFA lead to lipotoxicity and the development of metabolic disorders (31, 32).

Overall, WAT is involved in several physiological processes, as demonstrated by different studies on animal models. Studies on leptin-deficient ob/ob mice, with metabolic and immune dysfunctions, demonstrated that the transplantation of WAT normalizes high glucose levels, body weight and fertility (33), as well as thymus/spleen cellularity and inflammatory parameters like IL-6 (21). Moreover, a novel protective role for WAT in the immune response has been proposed (34). In particular, WAT from mice infected with bacteria represents a reservoir of memory T cell populations and promotes a protective memory response to infections (34). Studies in humans, and in particular in healthy centenarians, demonstrated that WAT becomes crucial in the extreme old age, as it secretes circulating factors such as adiponectin, that are associated with a protective metabolic and anti-inflammatory phenotype (35, 36).

As a whole, all these data suggest that WAT remodeling with aging may have not only negative but also positive effects on health.

### Brown Adipose Tissue (BAT)

BAT is a highly vascularized, heat-producing tissue, aimed at protecting animals from hypothermia through thermogenesis. This role is prominent in small size animals and newborns. It is distributed in cervical, supraclavicular, axillary, paravertebral, mediastinal, and upper abdominal regions (4). BAT is characterized by the presence of multilocular adipocytes containing abundant mitochondria that express high levels of UCP1 through which dissipate the proton gradient across the inner membrane and produce heat (37, 38). In addition to the traditional role of BAT in thermogenesis, recent data suggest that BAT plays an important endocrine role through the release of several endocrine factors, particularly in response to thermogenic activation (39). All these signaling molecules control metabolic processes via autocrine, paracrine, and endocrine mechanisms. These factors include (i) vascular endothelial growth factors (VEGFs) and insulin-like growth factor I (IGF-I), which, respectively, favor angiogenesis and increase the number of brown adipocyte precursor cells; (ii) several bone morphogenetic proteins (BMPs), implicated in the regulation of adipocyte differentiation and energy expenditure; (iii) thyroid hormone (triiodothyronine, T3), a well-recognized regulator of thermogenesis; iv. interleukin-6 (IL-6) and fibroblast growth factor 21 (FGF21). Although IL-6 is commonly considered a pro-inflammatory cytokine, studies of BAT transplantation demonstrate the beneficial effects of IL-6 derived from BAT in the control of metabolism (40). IL-6 is a key mediator to improve glucose homeostasis and insulin sensitivity, and contributes to the increase of circulating levels of FGF21 that plays an important role in the control of glucose and lipid metabolism (41).

In humans, BAT is abundant in newborns and infants, while it gradually declines from adolescence to adulthood (42, 43). In adults the amount of BAT is modulated by several factors, such as hormones, physical activity, cold exposure, and diet (44, 45), however the responsiveness to these stimuli declines during aging (46). Due to the endocrine role of BAT, several evidences demonstrate that its induction plays a protective role in counteracting age-related metabolic diseases. While the loss of BAT predisposes to WAT accumulation and weight gain (47), the transplantation of BAT in murine models induces an enhancement of energy expenditure, weight loss and insulin sensitivity, and prevents or even reverses obesity (48, 49). It was also shown that caloric restriction is able to stimulate BAT growth, conferring protection against the major age-related pathologies, such as cardiovascular diseases, cancer, and neurodegenerative disorders (50, 51). Moreover, increased levels of BAT mass promote longevity and enhance metabolism (52).

### Beige Adipose Tissue and Browning

BEIGE or BRITE (“brown in white”) adipose tissue is a subset of WAT with features of BAT. In particular, BEIGE tissue is composed of adipocytes derived from differentiation of WAT pre-adipocytes or trans-differentiation of WAT adipocytes, a phenomenon known as “WAT browning” (53, 54). In adults BEIGE tissue is localized within white fat depots, at inguinal and neck levels, where acts as WAT (55), while under particular stimuli, beige adipocytes acquire brown-like functions (56). The stimuli inducing the browning are not still well-understood, however it is known that they influence several molecular factors to orchestrate browning and thermogenesis mechanisms. Briefly, browning and thermogenesis are generally mediated by chronic cold exposure or hormones and peptide factors that activate the β-3-adrenergic receptor, an adipose tissue-selective adrenergic receptor (57). Moreover, the zinc finger protein PRDM16 and the mitochondrial protein UCP1 are considered the key contributors to prompt browning activity in the new BEIGE tissue (58). Noteworthy, a reduced expression of PRDM16 and UCP1 leads to the reversion of beige adipocytes into white adipocytes (59–61). Browning is a reversible mechanism pointing to the extraordinary plasticity of the adipose tissue. The process of browning is impaired during aging leading to a loss of BEIGE tissue and to a progressive decline in metabolic activity. This phenomenon is in part due to a reduction in the response to the β-adrenergic stimuli with aging (62). In fact, some studies have demonstrated that the loss of β-3-adrenergic receptor leads to an incapacity of white adipocytes to differentiate in beige adipocytes upon cold exposure (63, 64). Other studies in knockout or transgenic mice for different molecular regulators of browning underline the importance of this thermogenic tissue on age-associated metabolic dysregulations. As an example, in old mice the ablation of the winged helix factor FOXA3, a factor inducing the increase of adiposity and decrease of BAT mass with aging, induces browning, increases thermogenic capacity, decreases WAT expansion, thus leading to improved insulin sensitivity and lifespan extension (65). Other factors involved in the formation of brown adipocytes and in the regulation of lifespan are the BMPs. For example, BMP4, BMP7, and BMP8b control beige adipocyte development. In particular, mice treated with these factors increase browning processes and are protected from insulin resistance (29, 66).

### Ectopic Lipid Depots

Several tissues, such as bone marrow, skeletal muscle, liver and pancreas, make use of fatty acids as energy source, and these fatty acids accumulate within LDs in form of neutral lipids, mainly as triacylglycerols (TAGs) and steryl esters. LDs play an evolutionarily conserved role from yeasts to multicellular eukaryotic organisms (67). The physiological role of LDs is the maintenance of cellular energy homeostasis and the protection from lipotoxicity caused by an excess of FFA accumulation (68). An alteration in LD homeostasis leads to an excess of lipid intermediates, such as diacylglycerols, which in turn perturb metabolic pathways and cellular functions causing inflammation, mitochondrial stress and increase of reactive oxygen species (ROS) (68). The amount and size of LDs increase with age and contribute to the development of several age-related metabolic diseases such as type II diabetes, obesity, hepatic steatosis, and sarcopenia (68–75). Nevertheless, the primary causes of age-dependent ectopic fat accumulation remain largely unknown. However, not only the excess but also the deficiency of LDs leads to the onset of metabolic disorders (6, 20), in fact a growing body of evidence suggests that a balance between neutral lipid accumulation and their degradation is essential for the health and longevity of the organism. Numerous studies of model organisms, such as yeasts, nematodes, insects, and mice indicate that LDs are involved in the regulation of several longevity-related mechanisms, most of which are evolutionary conserved. It has been demonstrated that LDs control metabolic and lipid homeostasis and stimulate the release of different molecular mediators, such as lipophilic hormones that act as aging regulators and promote longevity (76). As an example, LD accumulation in the intestine of *Caenorhabditis elegans* contributes to the secretion of hormonal steroid pregnenolone, also present in humans, that extends lifespan (77–79). Consistently, the accumulation of TAGs has been reported as a novel pro-longevity factor in yeast. In fact, genetic manipulations leading to an increase in TAG content (by either the decrease of TAG lipases or the increase of TAG biosynthesis enzymes), extend yeast chronological lifespan (80, 81) independently of other lifespan regulators, such as dietary restriction (82). Moreover, LDs are involved in the adaptive stress responses and cell survival by the production of signaling molecules involved in the immune response pathway (83). As an example, in humans LDs serve as main storage site for arachidonic acid, which is the precursor of signaling molecules, such as eicosanoids or retinoic acids, which regulate inflammation (84).

All these findings indicate that the balance of fat accumulation in non-adipose tissues is important for the maintenance of a healthy status, in fact, both the excess and scarcity of fat are linked to the development of pathologies (20).

## The Endocrine Role of Adipose Tissue During Aging

Adipose tissue is an important endocrine organ that controls numerous physiological functions such as appetite, body weight, insulin sensitivity, fat distribution, glucose and lipid metabolism, neuroendocrine functions. This endocrine activity influences the whole body metabolism by releasing FFA, adipokines, cytokines and other molecular factors (85) that play a pleiotropic function on different tissues such as the liver, skeletal muscle, heart, lung, blood vessels, and sensory receptors, such as olfactory ones. Recent studies demonstrate that olfactory system is closely linked to adipose tissue in the regulation of energy balance (86, 87). In particular, the olfactory mucosa and bulb are provided with receptors for adipose-derived factors, such as leptin and adiponectin through which they influence body metabolism (88). How the age-related changes in adiposity modify olfactory function remains unclear. However, it is reasonable to think that the adipose tissue dysfunction with aging may affect energy homeostasis by perturbing the olfactory system through the production of these adipokines, and, in turn, outputs from the olfactory tract are among the causes of adipose tissue modifications.

WAT regulates also immune functions. In this regards, a link between white adiposity (in particular of VAT) and immune aging was found. In WAT, there are different types of immune cells that under stress conditions, such as obesity, impair their immune capacity increasing the pro-inflammatory behaviors in adipose tissue (89). Pro-inflammatory mediators can be produced also by senescent cells that accumulate in adipose tissue during aging (10). As for the olfactory system, the regulation of immune cell function by the adipose tissue depends on the secretion of adipokines such as leptin and adiponectin, that were originally classified as pro- or anti-inflammatory, respectively. The primary function of leptin is the regulation of appetite and energy expenditure by acting at the level of the central nervous system. It is known that higher circulating levels of leptin are present in obese and overfeeding individuals (90, 91). However, studies in ob/ob mice have demonstrated that the absence of leptin leads to obesity, hyperlipidaemia and insulin resistance, while leptin administration reverses these metabolic perturbations (92, 93). Accordingly, leptin administration has been proposed as a possible therapy to ameliorate glycemic control and dyslipidaemia in human patients affected by lipodystrophy or congenital leptin deficiency (94, 95). Likewise, adiponectin displays protective metabolic functions such as stimulating fatty acid oxidation and improving insulin sensitivity (96). High plasma levels of adiponectin are associated with low fat mass (97) and low incidence of metabolic disorders, including type II diabetes (98). Thus, leptin and adiponectin play different roles that converge on the maintenance of balanced energy levels and fat stores. Data from animal models highlight the critical functions of adipokines in metabolic homeostasis and longevity. In particular, these murine models, characterized by mutation or knockout in genes involved in metabolism, exhibit an extended lifespan associated to higher levels of plasma adiponectin, reduced adiposity, and lower-fasting insulin concentration (99–104). All these findings indicate that there is a clear association between elevated circulating levels of adiponectin and longevity, where the key mediator appears to be the maintenance of adiponectin-dependent insulin sensitivity. A recent cross-sectional study in caloric restricted mice of different age shows that longevity phenotype is linked to a specific adiponectin isoform with high molecular weight (105). This isoform was recognized as the most effective in enhancing the insulin sensitivity in humans (106). It is reported that the circulating levels of adiponectin and leptin (only in males), but not of resistin, increase with age (107). Noteworthy, also centenarians display higher levels of adiponectin associated to preserved insulin sensitivity. In particular, high adiponectin blood levels are associated with high levels of HDL-cholesterol and low levels of glycated hemoglobin (HbA1c) and C-reactive protein (35). Moreover elevated adiponectin levels are inversely correlated with the HOMA-IR, a risk factor for the onset of age-related metabolic diseases (108, 109).

Another factor secreted by adipose tissue is FGF21, an important metabolic regulator. FGF21 is in fact involved in the transcription of adiponectin in WAT, and these two factors together act to control energy metabolism and insulin sensitivity in other organs, such as liver and skeletal muscle (110, 111). FGF21 increases insulin sensitivity, energy expenditure, and weight loss also by inducing browning activity (112). Moreover, FGF21 has been also proposed as an antiaging hormone, in fact the overexpression of FGF21 in mice extends the lifespan (113). However, very recently we found that circulating levels of FGF21 in humans increase with age from 21 to 100+ years in healthy individuals and these high levels are related to worsened biochemical health parameters in old persons and decreased survival in extreme longevity (114). Accordingly, the role of FGF21 as a pro-longevity hormone has been questioned, as it appears to be responsible for the phenotype of accelerated aging in Opa1-deficient mice (115). This apparent contradiction could be explained in the framework of the hormetic paradigm. In other words, FGF21 is *per se* protective and likely able to recover a mild stress, but when the stress becomes stronger, it overcomes the beneficial effects of FGF21 and possibly an excessive production of FGF21 can have detrimental effects on the health status (114). WAT secretes also various bioactive compounds, indicated as Volatile Organic Compounds (VOCs) (116). VOCs are low-weight molecules produced by cellular metabolism that are involved in different physiological processes. VOCs mirror normal or abnormal physiological processes and reflect the metabolic condition of the organism and might have promise as diagnostic tools for a number of diseases as well as the metabolic condition of elderly people, as they seem to be characterized by a modified production of VOCs (117–119). Human fat can produce consistent amounts of VOCs detectable in breath, skin and sweat, and fluids, such as blood, urine, saliva, and in feces. It has been demonstrated that metabolic disorders or other diseases are characterized by specific VOCs profiles (120). Moreover, recent data demonstrate that the exposure to a new profile of VOCs contributes to an increase of pro-inflammatory cytokines involved in the development of metabolic diseases (116, 121). Therefore, it is reasonable to think that the age-related rearrangement of adipose tissue is at least in part responsible not only of the alteration of fat hormones production, but also of VOCs profiles, with at present unpredictable effects on the health status.

As a whole, adipose tissue and its derived adipokines have a critical role in controlling whole body energy metabolism, as well as some of the major age-related dysfunctions and longevity.

## Organ Involution and Fat Infiltration With Aging

One of the most universal manifestations of aging is the progressive atrophy or involution of many organs and tissues. This phenomenon is characterized by a loss of mass due to the concomitant reduction of cell proliferation and the increase of cell death rates, together with the accumulation of senescent cells (122). In some cases, this age-related loss of organ parenchyma does not lead to mere organ shrinkage, but is rather paralleled by an infiltration of adipocytes and sometimes by a complete replacement of organ with adipose tissue (122). The reasons why the age-related organ involution is accompanied by a replacement of adipose tissue remains poorly understood. As described above, adipose tissue has a crucial role in metabolic processes. Therefore, the adipose tissue substitution during organ involution is not a simple replacement with a neutral tissue, but it has to be considered as an adaptive response with dramatic biological consequences, as adipose tissue plays an active role as a source of hormones and adipokines that may regulate the homeostasis of the adjacent tissues (2, 91, 123). The substitution of parenchyma with adipose tissue is particularly evident in thymus, bone marrow, but also in muscle and pancreas, and generally begins between 20 and 50 years of age, excepting the thymic involution that begins much earlier, around late infancy-puberty (122). However, all human organs are gradually invaded by fat cells from birth onwards.

Thymus and bone marrow are two organs highly subjected to age-associated changes. They represent primary lymphoid organs, playing a fundamental role to provide cellular components of immune system during lifespan (124). The thymus is responsible for thymocyte differentiation and maturation into T cells, while bone marrow is the main site for the generation of all blood cells and for the maturation of B cells. The thymus progressively loses its functionality with age, in a process termed thymic involution or atrophy (125, 126). This phenomenon begins relatively soon after birth and results in a significant loss in thymic mass and a replacement with WAT within the thymus but also in the peripheral areas (127, 128). The organ replacement with WAT seems to occur in all species that possess the thymus indicating that this process is not only evolutionary ancient and conserved (129) but also important to counterbalance the loss of thymus by maintaining the size of this organ throughout life (125).

Aging induces adipocyte accumulation also in bone marrow cavities resulting in a loss of hemopoietic activity and bone loss disorders. Mesenchymal stem cells (MSC) of bone marrow can give rise to adipocytes or bone-forming osteoblasts. With age, these MSC tend to differentiate more into adipocytes that can become the prevalent cellular population of the marrow. Several studies suggest that this process leads to an impairment of osteogenic and hematopoietic activities (130–132) and is associated with the development of a great number of age-related diseases, such as osteoporosis and type II diabetes (133, 134). It is however suggested that the presence of adipose tissue in the marrow is necessary and beneficial for the health of the bone, especially when it has features of BAT (135).

As previously mentioned, aging is also associated with adipose tissue infiltration at non-adipose sites that normally are not involved in the fat storage. This age-related ectopic adiposity is associated with the progressive impairment of organs and tissues. This phenomenon affects, in particular, skeletal muscle, liver, pancreas, and heart. In skeletal muscle, elevated adipose deposition is observed at both intra- and inter-muscular sites. These two types of ectopic adiposity negatively impact on muscle strength and quality, as demonstrated in previously studies from our laboratory and others (75, 136, 137). In older people intra-muscular lipid content is associated with insulin resistance and the development of metabolic disorders (138). Moreover, in skeletal muscle, a subset of stem cells indicated as fibro-adipogenic progenitors (FAPs) appears to play a major role in inter-muscular adipose tissue (IMAT) infiltration, a phenomenon linked to progressive muscle dysfunction. FAPs appear to be driven toward adipogenic differentiation by muscle inactivity (139). It is therefore plausible to consider the increased adiposity of skeletal muscle observed with age as an adaptive response to mutated organismal requests.

Pancreas undergoes several alterations with aging, not only in volume but also structure. In particular, a significant reduction of pancreatic antero-posterior diameter, an increase of pancreatic lobulation and a decline of parenchymal component with a concomitant parenchymal fat mass increase (fatty replacement or lipomatosis) have been observed. This fatty replacement in pancreas is characterized by the infiltration of adipose tissue, as interlobular fat, between pancreatic lobules, accumulating around vessels (140, 141). The increase of pancreatic fat infiltration with aging is not well-understood and is still under debate. The degree of fatty replacement varies among subjects and depends on the condition, physiological, or pathological, in which each individual is involved. However, in any case, like in other organs, also the age-related atrophy of pancreas is associated to a replacement with fat mass.

In liver, an increase of intracellular fat mass in hepatocytes appears to be associated only with a progressive dysfunction of hepatic organ. Several evidence indicate that hepatic fat infiltration is associated with an increase of oxidative stress, inflammatory response, and cellular senescence, leading to the alteration of hepatic structure and the onset of non-alcoholic fatty liver diseases (NAFLD) (142, 143). Studies on the causes or effects of aging on hepatic adiposity are still limited. However, several evidence suggest that the progression of NAFLD is associated with telomere shortening, increased p21 expression and increased M1 macrophage inflammatory responses that are considered specific markers of cellular aging (144, 145). The increased expression levels of these markers are also observed in adipocytes, indicating that adipocytes under oxidative stress exhibit increased levels of ROS, shortened telomeres and switch to senescent/pro-inflammatory phenotype with the decline in insulin sensitivity (146).

In heart, the epicardial adiposity increases in size between myocardium and pericardium (147). In healthy conditions, cardiac adipose tissue has physiological functions, including metabolic, thermogenic and mechanical functions (148). New findings demonstrate that epicardial fat is mainly composed of adipocytes with features similar to brown or beige adipocytes (149), playing a protective role against the development of metabolic diseases (150). In particular, these adipocytes act through paracrine secretion of anti-atherogenic cytokines, such as adiponectin and adrenomedullin. However, the epicardial fat is susceptible to age-associated changes. In fact, with aging brown/beige adipose tissue undergoes a brown-to-white transition becoming dysfunctional and contributing to the onset of several pathologies.

**Figure 1:**
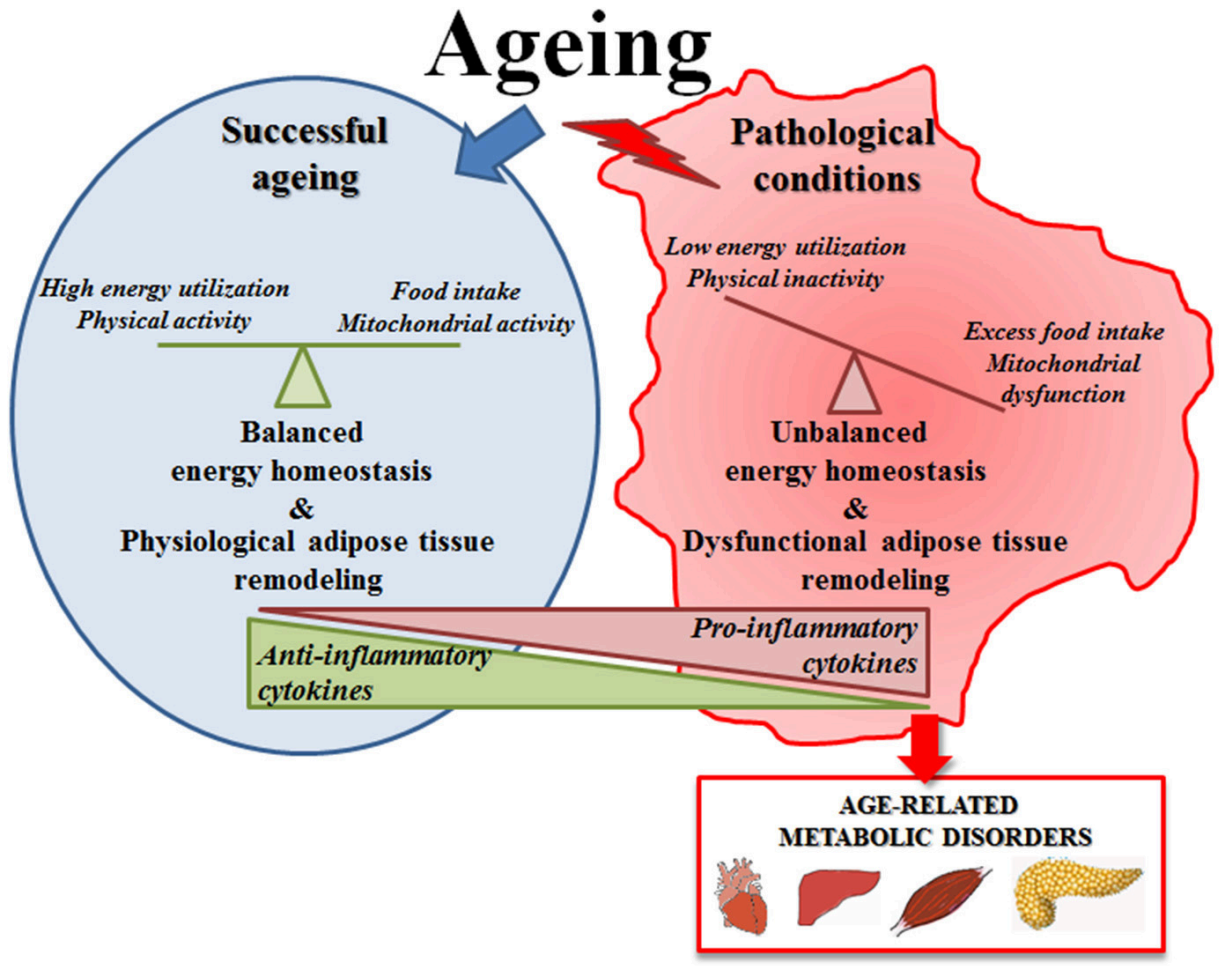
Schematic representation of adipose tissue function in successful aging or in pathological conditions. In successful aging a preserved balanced metabolism can maintain a physiological remodeling of adipose tissue that is likely an adaptive response to environmental conditions; in pathological conditions the unbalanced energy homeostasis leads to an excessive adipose tissue hypertrophy that becomes detrimental and contributes to the onset of age-related metabolic diseases.

All these data suggest that the increase of fat mass with age (including the increase of volume of adipose tissue, replacement of parenchyma and ectopic lipid deposition) is an apparently universal phenomenon, linked in general (but not exclusively) to the onset of pathological conditions (Figure 1). In fact, it is considered that adipocytes in adipose tissue do not change in number with age but rather in size, thus resembling the phenomenon occurring in obesity (151, 152). Adipocytes from obese persons are larger and unilocular, they release high amounts of FFA, produce less adiponectin and are infiltrated with M1 macrophages and produces pro-inflammatory cytokines (152), thus representing a risk factor for many age-associated diseases. The same seems to occur with age, as the large majority of the persons show a remodeling of adipose tissue with these features. In this regard, obesity could be considered as a sort of accelerated aging of the WAT. However, adipose tissue remodeling with age is likely an adaptive response to environmental conditions, therefore is not necessarily detrimental, and a preserved balanced metabolism can maintain a physiological remodeling of adipose tissue, without excessive WAT hypertrophy, but also without excessive loss of adiposity. This could be a key feature for achieving successful aging and longevity.

## Conclusions

The maintenance of a balanced amount of fat mass is crucial for health and survival, as discussed above. According to the “thrifty phenotype” theory, humans were selected to accumulate fat depots to face periods of food shortage. However, while a critical lower threshold of fat content exists, an upper threshold is apparently missing, and adipose tissue can accumulate in great amounts. The absence of an upper threshold for fat accumulation is probably due to the fact that this phenomenon did not occur in the wild frequently enough to undergo selection, or, alternatively, resulted neutral for the fitness of individuals.

**Figure 2:**
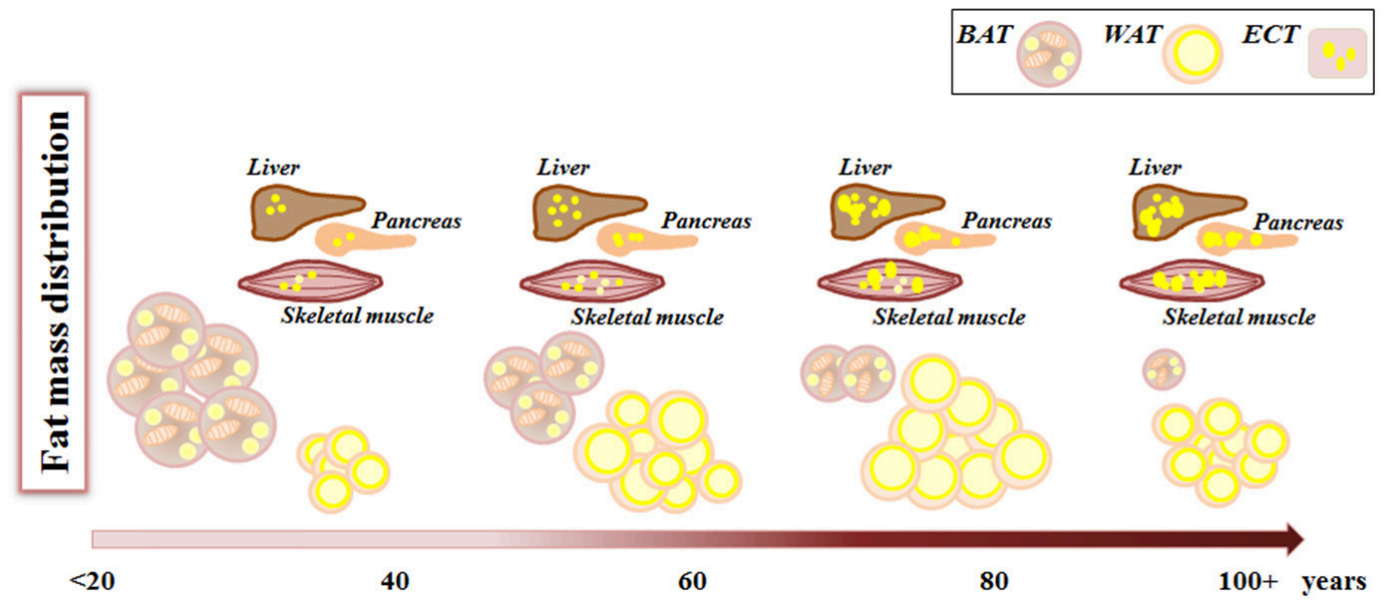
Fat mass remodeling with aging. During aging, from birth to advanced age, fat mass changes its amount and distribution. White adipose tissue (WAT) increases both in number and volume of adipocytes, while brown adipose tissue (BAT) decreases. Concomitantly there is an increase in lipid deposition in ectopic tissues (ECT) such as liver, muscles, pancreas where lipids are stored within lipid droplets.

With aging, the “thrifty phenotype” seems to emerge more dramatically, and the balance is tilted toward an increase of fat mass, at the level of VAT and SAT as well as in ectopic sites (liver, muscles, etc.) (Figure 2). This increase in fat deposition at the level of SAT and VAT can be considered an adaptive response to modified health conditions interacting with contingent environmental conditions, leading eventually to decreased energy expenditure. However, in some cases the storage of surplus energy can not be claimed as the reason for fat accumulation, especially when this occurs ectopically at the expenses of other tissue types with important vital functions, as in the case of thymic involution or skeletal muscle infiltration. In this case, it seems that fat deposition in form of WAT is a sort of physiological program (genetically determined?) for organs and tissues undergoing age-related atrophy or involution. We have mentioned the fact that different stem cell subpopulations such as muscle FAPs and bone marrow MSC preferentially differentiate to adipocyte with age, therefore, we are tempted to speculate that the pathway leading to this cell type is a sort of a default choice in involution processes. Should this speculation be verified, the reasons for this choice remain elusive.

The accumulation of WAT has been for long time viewed as detrimental, being the source of pro-inflammatory mediators and other important endocrine modulators and strongly associated with metabolic diseases such as insulin resistance and type II diabetes, cardiovascular diseases and cancer. However, it is not totally clear whether this negative role is present also in extreme old age. Actually, data on body composition in non-agenarians and centenarians are largely missing, even though the BMI of these people is usually lower than that of younger (70–80 years-old) persons. It is possible that, as for other risk factors like lipid serum profile and inflammatory parameters (153, 154), also the presence of a consistent amount of WAT can be important for survival at very advanced age. Further studies are needed to verify this hypothesis.

To sum up, human aging is characterized by a general tendency to increase adiposity, a phenomenon that in industrialized Countries can synergize with obesogenic conditions and become a health-threatening phenomenon. However, low adiposity is a risk factor too, as discussed, and a qualitatively adequate amount of fat is likely a key feature of long life.

## Author Contributions

MC, MM and SS: concept, analysis of literature, and writing; MS and CF: analysis of literature and critical discussion.

### Conflict of Interest Statement

The authors declare that the research was conducted in the absence of any commercial or financial relationships that could be construed as a potential conflict of interest. The handling editor declared a shared affiliation, though no other collaboration, with several of the authors MC, MM, CF, SS.
